# Kinsenoside inhibits the inflammatory mediator release in a type-II collagen induced arthritis mouse model by regulating the T cells responses

**DOI:** 10.1186/s12906-016-1054-8

**Published:** 2016-02-25

**Authors:** Hung-Bo Hsiao, Chang-Chi Hsieh, Jin-Bin Wu, Ho Lin, Wen-Chuan Lin

**Affiliations:** Department of Life Sciences, National Chung Hsing University, Taichung, Taiwan, ROC; Department of Animal Science and Biotechnology, Tunghai University, Taichung, Taiwan, ROC; School of Pharmacy, China Medical University, Taiwan. 91 Hsueh Shih Road, Taichung 404, Taiwan, ROC

**Keywords:** CIA, Kinsenoside, Rheumatoid arthritis, Inflammation

## Abstract

**Background:**

*Anoectochilus formosanus has* been used as a Chinese folk medicine and is known as the “King of medicine” in Chinese society due to its versatile pharmacological effects such as anti-hypertension, anti-diabetes, anti-heart disease, anti-lung and liver diseases, anti-nephritis and anti-Rheumatoid arthritis. Kinsenoside is an essential and active compound of *A. formosanus* (Orchidaceae). However, the anti-arthritic activity of kinsenoside has still not been demonstrated. In the present study, we confirmed that the kinsenoside treatment rheumatoid arthritis induced by collagen-induced arthritis in mice.

**Methods:**

Male DBA/1 J mice were immunized by intradermal injection of 100 μg of type II collagen in CFA. Kinsenoside was administered orally at a dose of 100 and 300 mg/kg once a day after 2nd booster injection. Paw swelling, arthritic score and histological change were measured. ELISA was used to measure cytokines including tumor necrosis factor alpha (TNF-α), interleukin-10 (IL-10), interleukin-17 (IL-17) and interferon-γ (IFN-γ) in the splenocyte according to the manufacturer’s instructions.

**Results:**

Compared with model group, kinsenoside significantly inhibited paw edema and decreased the arthritis score and disease incidence. Histopathological examination demonstrated that kinsenoside effectively protected bone and cartilage of knee joint from erosion, lesion and deformation versus those from the CIA group. Kinsenoside also decreased IL-1β, TNF-α, and MMP-9 expression, and increased the expression of IL-10 in inflamed joints. The administration of kinsenoside significantly suppressed levels of TNF-α, IFN-γ, and IL-17, but increased concentrations of IL-10 in the supernatants of each of the splenocytes in CIA mice compared with that in the H_2_O-treated mice with CIA. Using flow cytometric analysis, we demonstrated that kinsenoside increases the population of CD4^+^CD25^+^ regulatory T cells, thereby inhibiting the Th1 cell and B cell populations. Anticollagen IgG1 and IgG2a levels decreased in the serum of kinsenoside-treated mice.

**Conclusions:**

These results suggest that the administration of kinsenoside effectively suppressed inflammatory mediators’ production and bone erosion in mice with collagen-induced arthritis showing the potential as an anti-arthritis agent.

## Background

Rheumatoid arthritis (RA) is a chronic inflammatory disease, characterized by the occurrence of inflammatory synovitis, which leads to the destruction of cartilage and bone within joints through inflammatory cells that migrate to the synovial and periarticular tissues [1]. In states of chronic inflammation, such as in RA, the imbalance between proinflammatory and antiinflammatory cytokines determines the degree and extent of inflammation causing cellular damage [2]. Proinflammatory cytokines, including TNF-α, IL-17, IL-6, and IFN-γ, are the main mediators that initiate and cause inflammation to persist in RA, whereas antiinflammatory cytokines such as IL-4 and IL-10 can inhibit these processes [3]. Researchers have hypothesized that early inflammatory T cells, such as Th1 cells, play a critical role in the development of RA [1], and that Th1 cells (except for Th17 cells) are predominant in the joints of patients with RA, whereas Th2 cells, which suppress antigen-specific immune responses, are decreased [4]. Recently, the focus of research has expanded from Th1 and Th2 cells to regulatory T cells (Tregs), which can maintain immune homeostasis [5]. Current treatment modalities for RA either provide symptomatic relief (nonsteroidal antiinflammatory drugs; NSAIDs) or modify the disease process (disease-modifying antirheumatic drugs; DMARDs). According to the guidelines of the American College of Rheumatology, patients with newly diagnosed RA are strongly recommended to begin dual treatment by using NSAIDs to relieve nociceptive pain and control inflammation, and DMARDs to reduce disease activity, prevent joint deformity, and improve joint function [6]. The administration of these drugs is limited by the severe adverse effects of the drugs, such as gastrointestinal lesions, cardiovascular complications, and reproductive toxicity [7]. Therefore, researchers are focusing increasingly on plant-derived anti-RA drugs with high efficacy and few side effects. Recent studies have estimated that 60–90 % of patients with RA are highly likely to use botanical products [8]. This growing interest in alternative medical practices clearly indicates the necessity for more safe and effective anti-RA botanicals used in traditional medicine.

*Anoectochilus formosanus has* been used as a Chinese folk medicine and is known as the “King of medicine” in Chinese society due to its versatile pharmacological effects such as anti-hypertension, anti-diabetes, anti-heart disease, anti-lung and liver diseases, anti-nephritis and anti-Rheumatoid arthritis [9]. Therefore, *A. formosanus* extract is often used as a health supplement in Taiwan. Kinsenoside [3-(*R*)-β-D-glucopyranosyloxybutanolide] is an essential and active compound of *A. formosanus* (Orchidaceae) [10]. In previous studies, we discovered that kinsenoside ameliorated lipopolysaccharide (LPS)-induced shock in mice, and carbon tetrachloride caused hepatitis in mice by inhibiting macrophage activation [11, 12]. Kinsenoside significantly inhibited the LPS-induced production of nitric oxide in both peritoneal lavage macrophages and RAW 264.7 cells in mice by suppressing NF-κB activation [11]. In addition, we discovered that a standardized aqueous extract of *A. formosanus* modulated Tregs, thereby increasing the immunosuppression of airway hyperresponsiveness in mice that inhaled ovalbumin [13]. Therefore, kinsenoside may be able to downregulate both autoimmune and inflammatory responses in mice with collagen-induced arthritis (CIA). Although as far as we know, no studies have focused on the in vivo effects of kinsenoside. We encourage researchers to evaluate the anti-arthritic effects of kinsenoside in future studies.

In this study, we evaluated the immunomodulatory role of Tregs following the administration of kinsenoside during the initiation and establishment of arthritis in mice with CIA. Furthermore, we discussed the immunomodulatory role of Tregs following the administration of kinsenoside in proinflammatory cytokine release, and the resulting decrease in matrix metalloproteinases (MMP)-9 expression.

## Methods

### Animals

Male DBA/1 J mice were obtained from the Jackson Laboratory (Bar Harbor, Maine, USA), and were housed in standard laboratory cages and allowed access to tap water *ad libitum*. The experimental animals were housed in an air-conditioned room at 22–25 °C under a 12 h light–dark cycle. All animals were treated in accordance with the Institutional Animal Care and Use Committee (IACUC) of China Medical University, and the study protocol was approved by the ethics committee of the China Medical University, Taichung, Taiwan.

### Preparation of kinsenoside

Kinsenoside was prepared by Associate Professor Wu. Fresh whole plants of A. formosanus (10 kg) were extracted with water and the filtrate was partitioned successively with ethyl acetate. Water-soluble portions (AFEW) were evaporated under reduced pressure, yielding 218.4 g of red residue. AFEW (210 g) was applied to a DIAION HP-20 column (Nippon Ressui Co., Japan) and eluted with H_2_O, 10, 20, and 50 % methanol in water, and 100 % methanol to provide five fractions (AFEW-1–AFEW-5). The dry weight of fraction AFEW-2 was 22.1 g. Fraction AFEW-2 (10 g) was further purified using silica gel (Si 60 F245; Merck, Germany) with chloroform/ethanol (15:8) as the mobile phase to provide four fractions. Fraction 4 (4.5 g) was applied to preparative high-performance liquid chromatography (HPLC) to yield a pure compound (4.1 g). Conditions used for the preparation of HPLC were as follows: pump, Shimadzu LC-8A (Kyoto, Japan); mobile phase, water; column, Mightysil ODS RP-18 Aqua column (i.d. 20 mm, 250 mm long; 5 μm particle size; Kanto Chemical Co., Tokyo, Japan). The pure compound was identified by mass spectroscopy (Jeol GCmate, Tokyo, Japan). The content of kinsenoside was measured using HPLC. The conditions of HPLC were the same as those described in a previous study [14]. The identity and purity of the kinsenoside (>85 %) was analyzed using high-performance liquid chromatography based on a previous study [14].

### Induction and assessment of collagen-induced arthritis

CIA is typically induced using intradermal immunization with type II collagen (CII) in Freund’s complete adjuvant (CFA, Sigma-Aldrich, St. Louis, MO, USA), followed by an intradermal booster immunization after 21 days [15]. In brief, CII purchased from Sigma-Aldrich (St. Louis, MO, USA) was reconstituted at 2 mg/ml in 50 mM acetic acid. Mice received an intradermal injection of 100 μg of CII in CFA (Day 0, primary immunization), and a booster injection of 100 μg of CII in Freund’s incomplete adjuvant (Sigma-Aldrich, St. Louis, MO, USA) on Day 21 (2nd booster injection). Following a prophylactic dosing program, the animals were randomly divided into four experimental groups, each containing six individuals, and treated follows:

- Naive: no treat, normal.
- CIA control: H_2_O 1 ml/kg *p.o.*
- CIA+ kinsenoside 100 mg/kg: kinsenoside 100 mg/kg *p.o.*
- CIA+ kinsenoside 300 mg/kg: kinsenoside 300 mg/kg *p.o.*

The mice were orally administered kinsenoside (100 or 300 mg/kg in water) daily, from Day 1 to Day 21 after 2nd booster injection, and monitored for disease incidence and severity up to Day 21. The CIA control mice were administered water alone.

Arthritic severity scores were derived as follows: The clinical scores for paws were used to reflect the severity of arthritis, and were classified as 0 (normal joints), 1 (swelling in one digit or joint inflammation), 2 (swelling in two or three digits, or slight paw swelling), 3 (swelling in more than four digits and moderate swelling in the entire paw), and 4 (severe swelling and deformation of the paw, also presenting rigid joints); the sum of the scores of all four paws of each mouse represented the total clinical score. Cumulative disease scores were calculated by adding the daily disease scores from the day following immunization onward, until the end of the experiment. The incidence of arthritic paws was defined as the occurrence of inflamed paws with a clinical arthritis score of 2 or higher among four paws.

On Day 21, all of the mice were anesthetized and blood was collected using cardiac puncture; the mice were then sacrificed using cervical dislocation after 2nd booster injection. The mice spleens were removed and used for enzyme-linked immunosorbent assay (ELISA) analysis, total cell count, flow-cytometric analysis, and histological and histochemical examinations.

### Identification of anticollagen antibodies in serum

Identification of anticollagen antibodies in serum followed according to the procedure of Poosarla et al. (2011) [16]. Serum collected at the end of the experiment was stored at − 80 °C. The level of anti-CII antibodies was measured using ELISA analysis. In brief, 200 μl/well of CII (10 μg/ml) in phosphate-buffered saline (PBS) was coated on plates at 4 °C, and left to stand overnight. After washing, the plates were treated with blocking buffer solution (50 mM Tris, 0.14 M NaCl, 1 % BSA, pH 8.0) for 30 min. After washing the plates, the samples (100 μl/well) were incubated for 90 min at room temperature. After washing the plates,, the plates were incubated with horseradish-peroxidase (HRP)-conjugated goat antimouse IgG1, or IgG2a (Bethyl Laboratories, Inc., Montgomery, TX, USA) for 60 min. After washing, the plates were placed in tetramethylbenzidine (TMB; Kirkegaard & Perry Laboratories, Gaithersburg, MD, USA) substrate solution, and incubated at room temperature for 30 min. The enzymatic reaction was terminated by adding 50 μl of stop solution (2 M H_2_SO_4_), and the absorbance of each well was measured at 450 nm (TRIAD multimode reader, Dynex Technologies, Sullyfield Circle, USA).

### CII-induced cytokine production

Splenocytes were collected from all groups, both with and without CIA immunization (*n* = 6 for all groups) on Day 21 of the prophylactic protocol, and seeded with 1 × 10^6^ cells/well in RPMI-1640 supplemented with 10 % FBS, and cultured for 48 h with or without 5.0 μg/ml Con A (Sigma-Aldrich St. Louis, MO, USA) in 24-well plates. Supernatants were collected and stored at − 80 °C. Cytkine concentrations, including TNF-α, IFN-γ, IL-10 (eBioscience, San Diego, CA, USA), and IL-17 (R&D Systems Inc., Minneapolis, MN, USA), were measured using by ELISA according to the manufacturer’s protocol

### Gene expression analysis

RNA was extracted from the left hind paws of the mice, snap-frozen in liquid nitrogen, ground into powder, and homogenized. This procedure was conducted under RNase-free conditions. Total RNA was extracted from the tissue homogenates by using TRIzol (Invitrogen; Carlsbad, CA, USA) according to the manufacturer instructions. The total RNA (3 μg) was subjected to room temperature reaction by using Moloney murine leukemia virus reverse transcriptase (M-MuLV, Hopegen Biotechnology, Taichung, Taiwan, ROC) according to the directions in the instruction manual. The specific primers are listed in Table 1. The cDNA was amplified with a combination of sense and antisense primer pairs and PCR master mix (Fermentas International Inc., Burlington, Canada) by using a thermal cycler (Astec, Tokyo, Japan).

**Table 1:** PCR primers

Gene name	primers	bp	cycle	(°C)
IL-10	F:CCCTTTGCTATGGTGTCCTT	97	35	50
	R:TGGTTTCTCTTCCCAAGACC			
IL-1β	F:TTCAAGGGGACATTAGGCAG	158	35	51
	R:TGTGCTGGTGCTTCATTCAT			
TNF-α	F:CTCAGCGAGGACAGCAAGG	108	35	53
	R:AGGGACAGAACCTGCCTGG			
MCP-1	F:AGAGAGCCAGACGGGAGGAA	190	35	56
	R:GTAGCAGCAGGTGAGTGGGG			
MMP-9	F:GGTCTAGGCCCAGAGGTA	310	35	57
	R:GGTCGTAGGTCACGTAGC			
T-bet	F:TTCAACCAGCACCAGACAGA	109	35	62
	R:ACATCCTGTAATGGCTTGTGG			
GATA-3	F:TTCAAGGGGACATTAGGCAG	78	35	60
	R:TGGTGGTGGTCTGACAGTTC			
GAPDH	F:CTTCATTGACCTCAACTACATGGTCTA	99	35	55
	R: GATGA CAAGC TTCCC ATTCT CAG			

### Flow cytometric analysis

Splenocytes were collected for analysis by using flow cytometry. Single-cell suspensions, both with and without CIA, were identified by detecting surface markers with the specific binding of monoclonal antibodies (mAb). In brief, 50 μl of cell suspension (5 × 10^5^ cells) was incubated in the presence of saturating concentrations of fluorescein-, phycoerythrin-, or PE-Cy5-conjugated mAb (eBioscience, San Diego, CA., USA) on ice for 30 min in darkness. Cytofluorometric analysis of the lymphocyte fractions was performed using side scatter and forward scatter with laser excitation at 488 nm. Flow cytometric data acquisition and analysis of cell populations of at least 1 × 10^4^ leukocytes were performed on a CellQuest computer system (BD Biosciences). Percentage list mode data were calculated based on the number of lymphocytes identified in each quadrant. B cells were determined using the surface markers CD19^+^ (clone MB19-1) and CD45^+^ (clone 30-F11), T helper 1 (Th1) cells were determined using CD4^+^ (clone RM4–5) and Tim-3^+^ (clone 8B.2C12), and Tregs were determined using CD4^+^ and CD25^+^ (clone 7D4).

### Histological and histochemical examination

On Day 21, the left hind paws of the H_2_O-treated group, the kinsenoside-treated group, and the control group were fixed in 10 % neutral phosphate-buffered formalin. These specimens were decalcified in 10 % EDTA for 2 weeks, embedded in paraffin, and sectioned at 5-μm intervals. Hematoxylin and eosin staining, tartrate-resistant acid phosphatase (TRAP) staining, and immunohistochemical staining were performed by following standard methods. For the TRAP assay of osteoclast activity, samples were embedded in paraffin and prepared for TRAP staining. After washing with distilled water, samples were incubated for 60 min at 37 °C in darkness in a solution containing Fast Garnet GBC, sodium nitrite, napthol AS-BI phosphoric acid, acetate, and tartrate, which was made using a Leukocyte Acid Phosphatase Assay kit (Sigma-Aldrich, St. Louis, MO). The samples were washed with water, and TRAP-positive multinucleated cells, containing three or more nuclei, were counted under a microscope. For immunohistochemical staining, the cryostat section of joints was fixed in cold acetone for 10 min, washed in PBS, and treated with 0.3 % H_2_O_2_ in absolute methanol for 15 min to deplete endogenous peroxidase. After blocking nonspecific binding by using 10 % nonfat milk in PBS for 30 min, the sections were incubated with antirabbit MMP-9 Ab (Millipore, MA, USA) or CD 68^+^ Ab (Abcam, Cambridge, UK) at appropriate dilutions for 1 h at room temperature, washed, incubated with biotinylated antirabbit IgG, washed and incubated with avidin-biotinylated HRP complex and diaminobenzidine tetrahydrochloride (Elite kit; Vector Laboratories Inc., Burlingame, CA, USA), and counterstained with Mayer’s hematoxylin.

### Splenocyte preparation and CD4^+^ T cell isolation

Splenocytes were prepared by disrupting the spleen between glass slides in complete medium (RPMI 1640 with 10 % FBS and 1 % PSA). After 10 min of centrifugation at 300 × *g* to separate cells from debris, the cells were washed in RPMI medium, followed by the lysis of erythrocytes by using 0.1X HBSS and 2X HBSS. Splenic CD4^+^ cells were purified using flow cytometry (BD FACSAria) to perform positive selection with CD4^+^ (eBioscience, San Diego, CA., USA). The purity was >95 %.

#### In vitro Th1 and Th2 cell polarization

CD4^+^ T cells (1 × 10^6^ cells/well) were resuspended in complete medium (RPMI 1640 with 10 % FBS and 1 % PSA) and activated with plate-bound 6 μg/ml anti-CD3 (BioLegend, San Diego, CA) and 6 μg/ml anti-CD28 (BioLegend, San Diego, CA) for 24 h. Naive CD4^+^ T cells were incubated with 20 ng/ml rIL-12 (BioLegend, San Diego, CA), 20 μg/ml anti-IL-4 (BioLegend, San Diego, CA), and 20 ng/ml rIL-2 (ProSpec-Tany TechnoGene Ltd., Rehovot Science Park, Israel) for Th1 differentiation. For Th2 cell differentiation, 20 μg/ml IL-4 (ProSpec-Tany TechnoGene Ltd., Rehovot Science Park, Israel) and 10 ng/ml rIL-2 (ProSpec-Tany TechnoGene Ltd., Rehovot Science Park, Israel) were added to the culture medium in the presence of 20 ng/ml rIL-2. Cells were cultured for 7 days and harvested for the preparation of total RNA.

### Statistical analysis

Results are expressed as the mean ± standard deviation [17]. All experimental data were analyzed using one-way analysis of variance following the Dunnett test. *P* < 0.05 was considered statistically significant. The Mann—Whitney *U* test was used to determine the severity scores of the kinsenoside group and the H_2_O-treated CIA group, and the scores were expressed as the mean ± SD for each group at each time point. *P* < 0.05 was considered statistically significant.

## Results

### Effects of kinsenoside on mice with collagen-induced arthritis

**Fig. 1:**
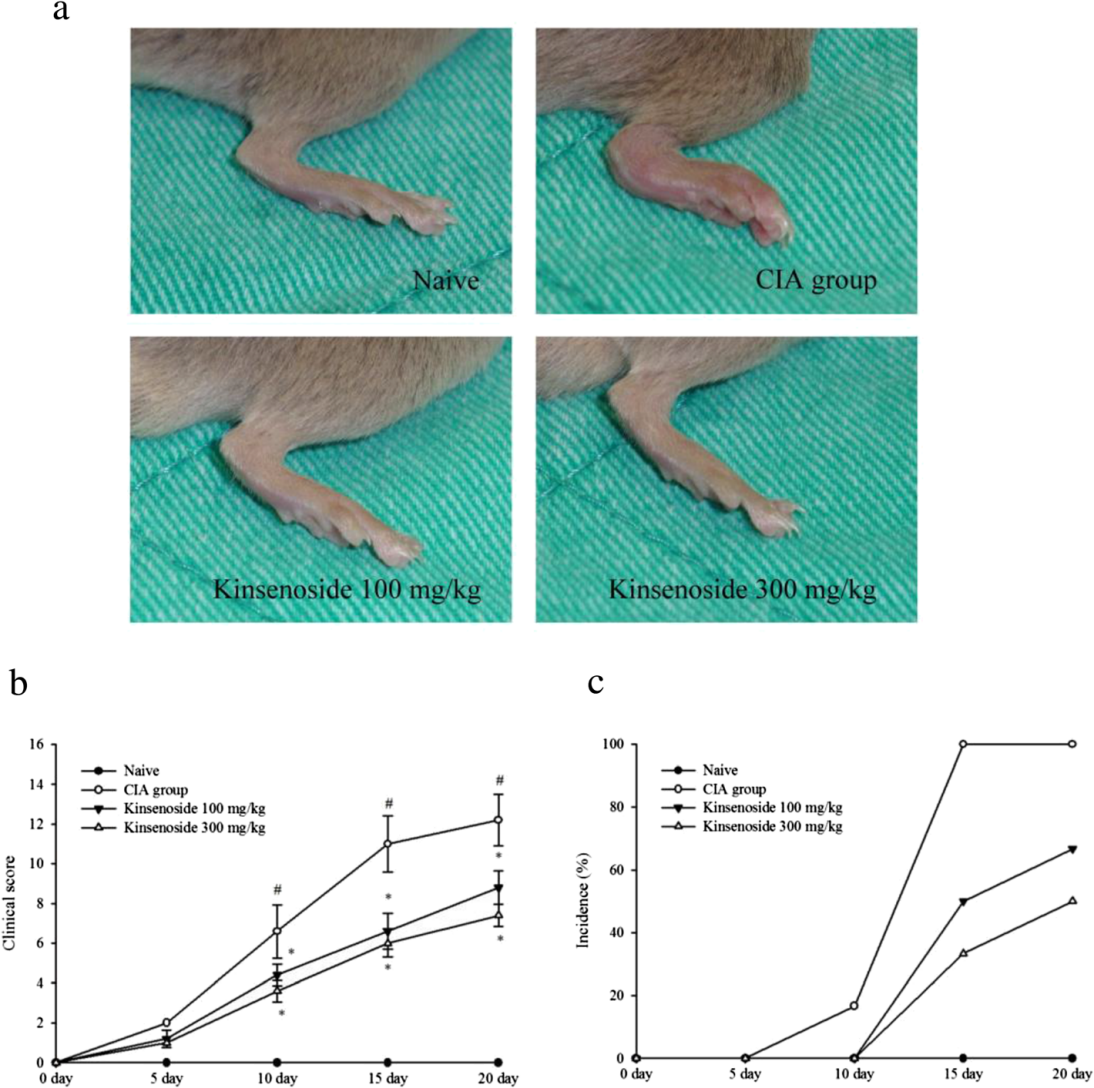
The effects of kinsenoside on the development and clinical of CIA. **a** Photograph type (hind paw volume). (**b**). Clinical scores of CIA were monitored every 5 day after booster immunization. (**c**)The incidence of arthritic paws overtime was monitored. (*p < 0.05 vs. H_2_O-treated group, *n* = 6 in each group)

We used a mouse CIA model to determine whether kinsenoside exerts suppressive effects on RA. Kinsenoside administered daily significantly suppressed CIA in mice in a dose-dependent manner. Mice treated with 100 and 300 mg/kg of kinsenoside exhibited significant reductions in incidence and severity of CIA (Fig. 1a). On Day 10, arthritis incidence and mean severity score for the group treated with 300 mg/kg of kinsenoside were 3.6 ± 0.5, respectively, as opposed to 6.6 ± 1.3 for the H_2_O-treated group. This treatment significantly reduced arthritis severity scores on 0–20 day and arthritis scores and disease incidence attained their maxima after 2nd booster injection in all mouse groups (Fig. 1b and c).`

### Effects of kinsenoside on joint histology

**Fig. 2:**
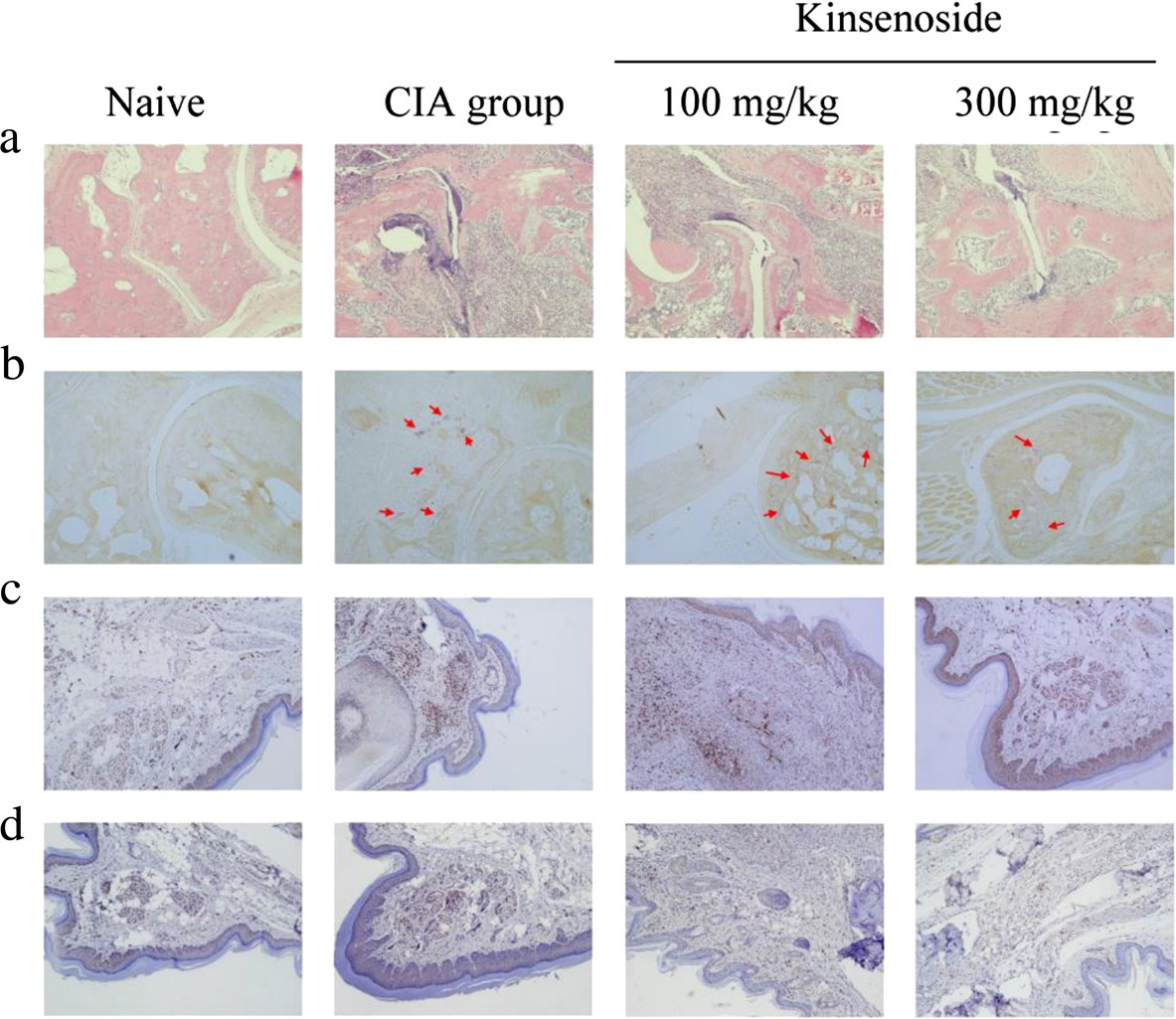
Pathologic morphology of the knee joints following administration of kinsenoside to mice after the onset of CIA. H&E staining **a** TRAP (**b**) and immunohistochemical staining MMP-9 (**c**) and CD 68^+^ (**d**) sections of the synovial tissues from mice with Naïve and CIA treated with H_2_O and kinsenoside. Original magnification × 200

To investigate the effects of kinsenoside on the progression of CIA, the oral administration of kinsenoside (100 and 300 mg/kg) was initiated 1 day after 2nd booster injection. After sacrificing the mice, we evaluated the histology of the synovial tissues. Figure 2a illustrates that kinsenoside inhibited cartilage and bone destruction, as well as inflammatory responses in the treated group, compared with that in the H_2_O-treated CIA group. The treatment of mice with CIA by using kinsenoside resulted in significant reductions in cellular infiltration, pannus formation, and the destruction of cartilage and bone in arthritic joints, with low pathogenic scores.

TRAP+ osteoclasts (red staining) were present on the bone surfaces of the mice with CIA not treated with kinsenoside, particularly around the navicular bone. In the kinsenoside-treated mice, however, the accumulation of TRAP+ cells was significantly less than that in the mice not treated with kinsenoside, and navicular bone structure was maintained (Fig. 2b).

Immunohistochemical staining of synovial tissues in the H_2_O-treated CIA group revealed high levels of MMP-9 and CD68^+^ expression. By contrast, mice with CIA treated with kinsenoside exhibited significant reductions in MMP-9 and CD68^+^ expression (Fig. 2c and d).

### Kinsenoside inhibits inflammatory cytokines and MMP-9 expression in the joints of mice with collagen-induced arthritis

**Fig. 3:**
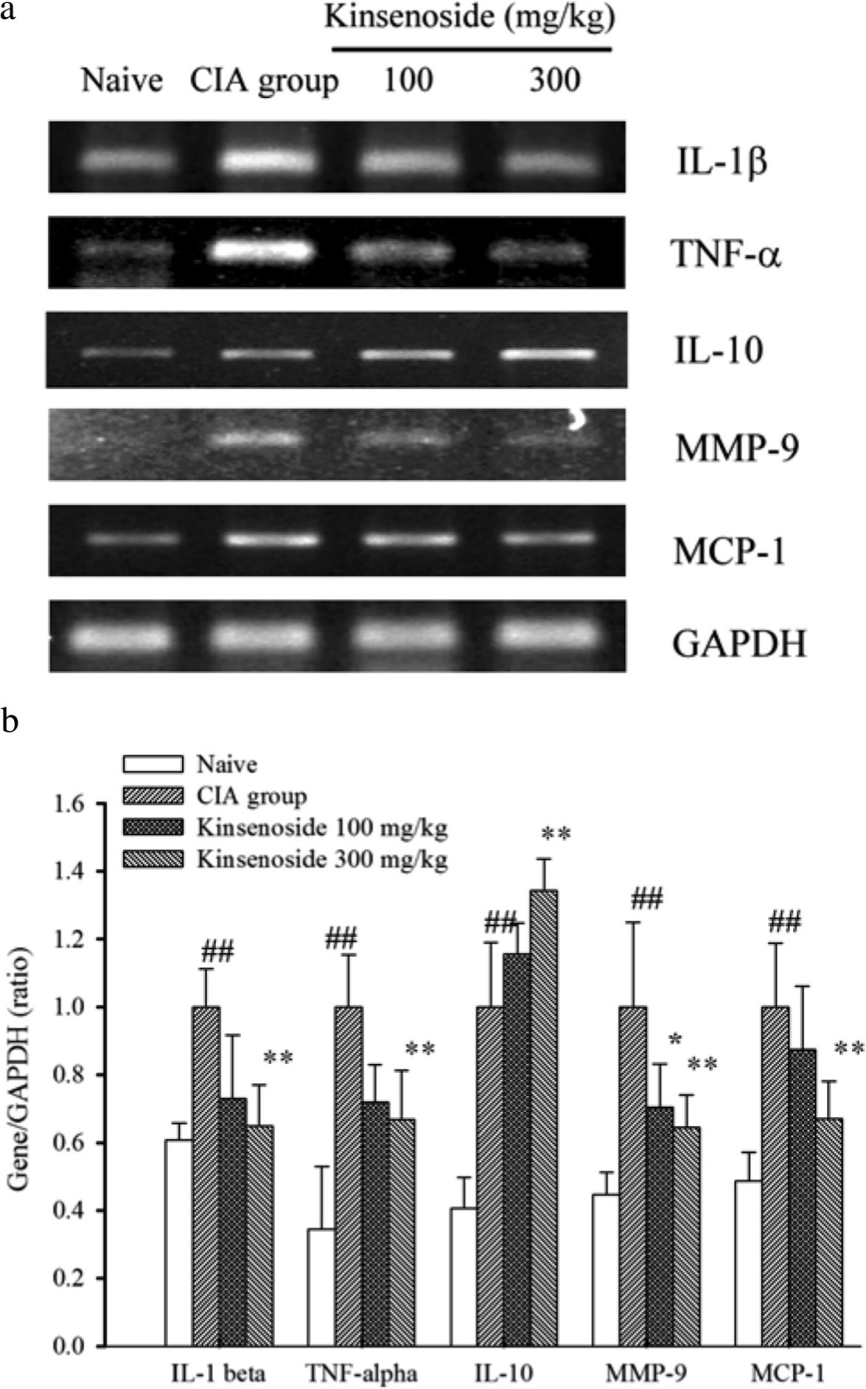
Effects of kinsenoside on mRNA expression of the inflammatory cytokines, *MMP-9* and *MCP-1* in the joints of CIA mice. Fragments were amplified by RT-PCR (**a**). The expression levels of IL-1β, TNF-α, IL-10, MMP-9 and mRNA were measured and quantified densitometrically (**b**). The mice were treated as described in the Materials and methods section. Values were expressed as means ± SD, *n* = 6 in each group. *# P* < 0.05, *## P* < 0.01 as compared with the naïve group. * *P* < 0.05, ** *P* < 0.01 as compared with the H_2_O-treated group

Local and circulating cytokine levels were measured in the joint tissues and sera to obtain insight into the mechanisms of beneficial kinsenoside-mediated effects. In Fig. 3, the reverse-transcription polymerase chain reaction (RT-PCR) results indicated the presence of relatively small amounts of IL-1β, TNF-α, and MMP-9 mRNA in the joints of healthy mice. However, the concentrations of these transcripts exhibited a substantial increase in the joints of the mice with CIA. Kinsenoside treatment significantly reduced elevated levels of IL-1β, TNF-γ and MMP-9. By contrast, the expression of IL-10 in the joints of mice with CIA was significantly lower than that of healthy mice (Fig. 3), and the administration of 300 mg/kg of kinsenoside increased IL-10 gene expression to levels comparable with those of the H_2_O-treated group. These results suggested that kinsenoside prevented the destruction of connective tissues by simultaneously augmenting IL-10 production and reducing IL-1β, TNF-α, and MMP-9 production at inflammatory sites in mice with RA.

### Kinsenoside downregulates proinflammatory cytokine production

**Fig. 4:**
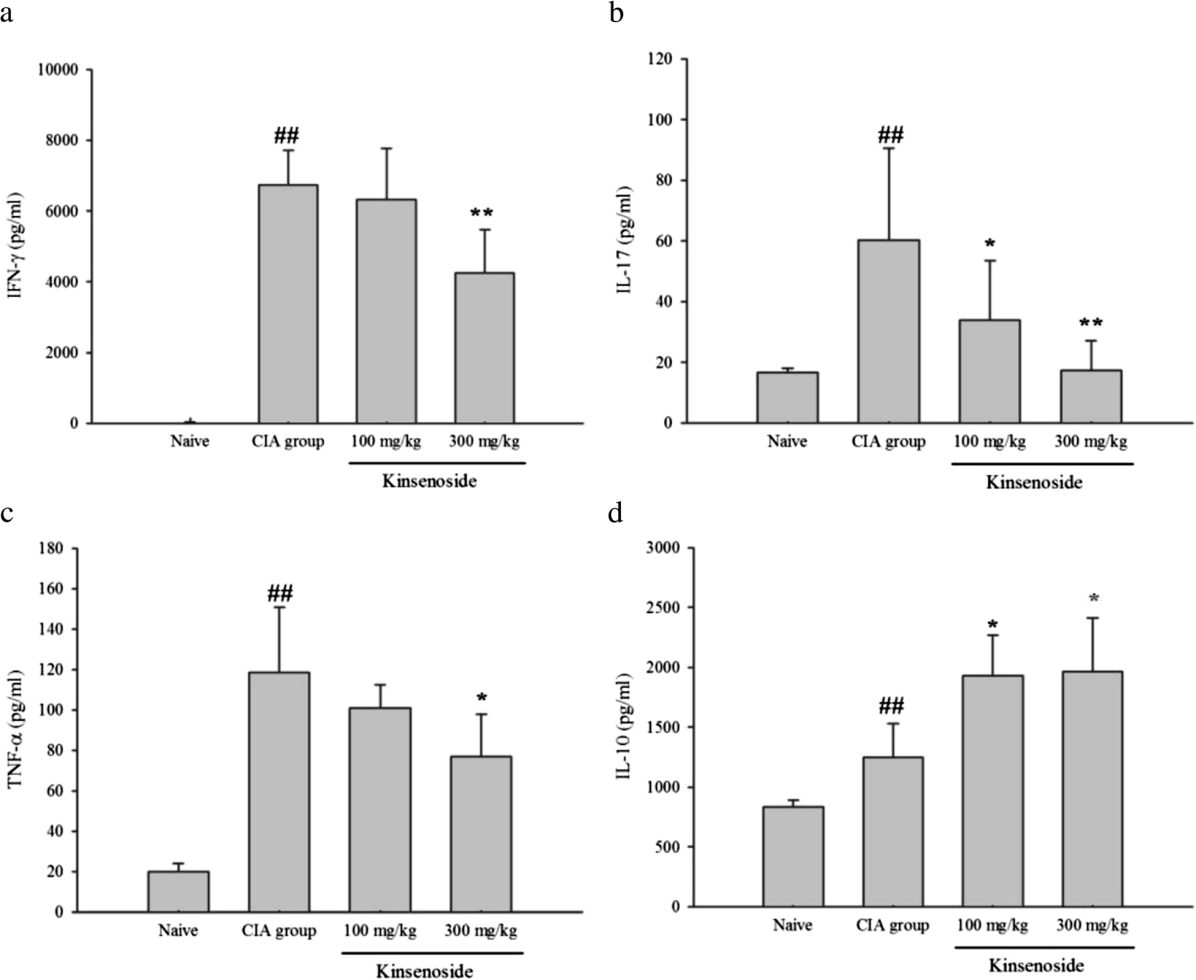
Inhibitory effect of kinsenoside on CII-induced cytokine production by splenocytes in vitro. Splenocytes from normal control or CIA mice were cultured for 48 h with or without 50 μg/ml Con A. The supernatants were collected, and the IFN-γ **a** IL-17 (**b**), TNF-α (**c**) and IL-10 (**d**) levels were measured. Values were expressed as means ± SD, *n* = 6 in each group. *# P < 0.05, ## P < 0.01 *as compared with the naïve group. * P < 0.05, ** P < 0.01 as compared with the H_2_O-treated group

We performed in vitro assay of CIA-induced cytokine production by mouse splenocytes to clarify whether kinsenoside treatment regulates cytokine production. Equivalent numbers of splenocytes from the H_2_O-treated and kinsenoside-treated mice with CIA were cultured with Con A. We measured the production of Th1-related cytokines (IFN-γ), Th2-related cytokines (IL-10), Th17-related cytokines (IL-17), and proinflammatory cytokines (TNF-α) in the supernatants of each splenocyte culture. We discovered that the levels of IFN-γ, IL-17, and TNF-α were significantly suppressed in the supernatants of each of the splenocyte cultures of kinsenoside-treated mice with CIA, compared with that in the H_2_O-treated mouse group (Fig. 4a, b, and c).

However, the levels of IL-10 in the splenocytes of the kinsenoside-treated mice were significantly increased compared with that in the H_2_O-treated mice (Fig. 4d). These data indicated that kinsenoside suppressed Con A-induced cytokine production (IFN-γ, IL-17 and TNF-α) in the supernatants of each of the splenocyte cultures during the progression of arthritis.

### Fluorescence-activated cell sorting analysis of splenocytes

We investigated the recruitment of total cells in splenocytes, and measured the effects of kinsenoside on inflammatory cells to evaluate the effects of kinsenoside on mice with CIA. The cell population of the splenocytes in the kinsenoside-treated group exhibited a decrease, compared with that in the H_2_O-treated controls (Table 2). Fluorescence-activated cell sorting analysis of CD4^+^, CD19^+^, CD25^+^, CD45^+^, and Tim-3^+^ cell populations, in both healthy mice and those with RA, revealed that the population of Th1 cells produced by CD4^+^Tim-3^+^ cells was higher in the splenocytes of the H_2_O-treated group than in those of the other groups. The CD19^+^CD45^+^ B cell population exhibited an increase in the H_2_O-treated group, but not in the kinsenoside-treated mice with CIA (Table 2). The Th1 cell population was lower in the kinsenoside-treated CIA group splenocytes (300 mg/kg) than in the H_2_O-treated group (Table 2). The population of Tregs among total CD4^+^CD25^+^ cells in the H_2_O-treated CIA group was lower than that in the control group, but higher than that in the kinsenoside-treated groups (Table 2).

**Table 2:** Effect of kinsenoside on the splenocytes in vivo

The splenocytes (%)	Control	H_2_O	Kinsenoside 100 mg/kg	Kinsenoside 300 mg/kg
CD4^+^Tim-3^+^ cells	4.96 ± 0.63	9.12 ± 0.79 ##	7.62 ± 0.93**	7.04 ± 0.74**
CD4^+^CD25^+^ cells	8.31 ± 0.48	6.21 ± 0.82 ##	7.82 ± 0.91**	8.26 ± 0.78**
CD19^+^CD45^+^ cells	26.99 ± 4.56	36.2 ± 2.52 ##	25.84 ± 5.64**	23.75 ± 3.98**

Population of the splenocytes in normal control and CIA mice. The cell populations of the CD4^+^Tim-3^+^, CD4^+^CD25^+^ and CD19^+^CD45^+^ cells in the splenocytes. Values were expressed as means ± SD, *n* = 6 in each group. ^*##*^ *P < 0.01* as compared with the control group. **P < 0.01 as compared with the H_2_O-treated group

### Kinsenoside downregulates Ag-specific B cell responses

**Fig. 5:**
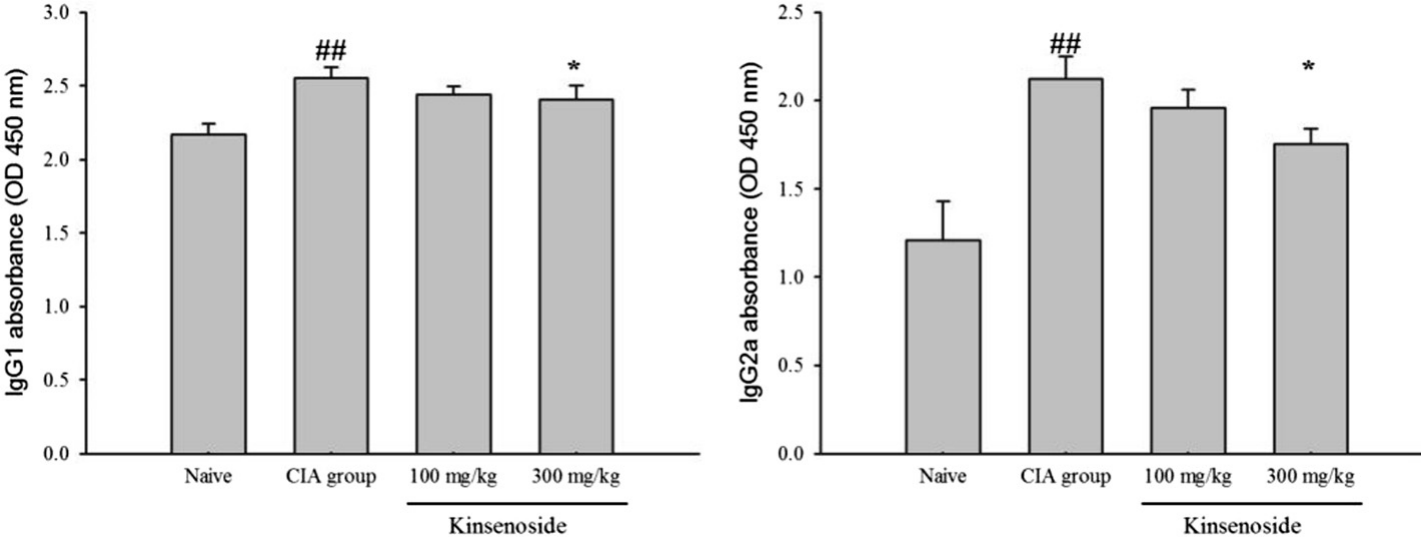
Effects of kinsenoside on anti-CII IgG1 and IgG2a. Serum was obtained from 6 mice of each group on day 21 after the booster injection. Values were expressed as means ± SD, *n* = 6 in each group. *#P < 0.05, ##P < 0.01* as compared with the naïve group. *P < 0.05, **P < 0.01as compared with the H_2_O-treated group

We assessed the levels of CII-specific IgG1 and IgG2a antibodies, and the kinsenoside-treated group exhibited downregulation of anti-CIA IgG1 and IgG2a antibody production (Fig. 5).

### Effects of kinsenoside on the modulation of Th1/Th2 cell polarization

**Fig. 6:**
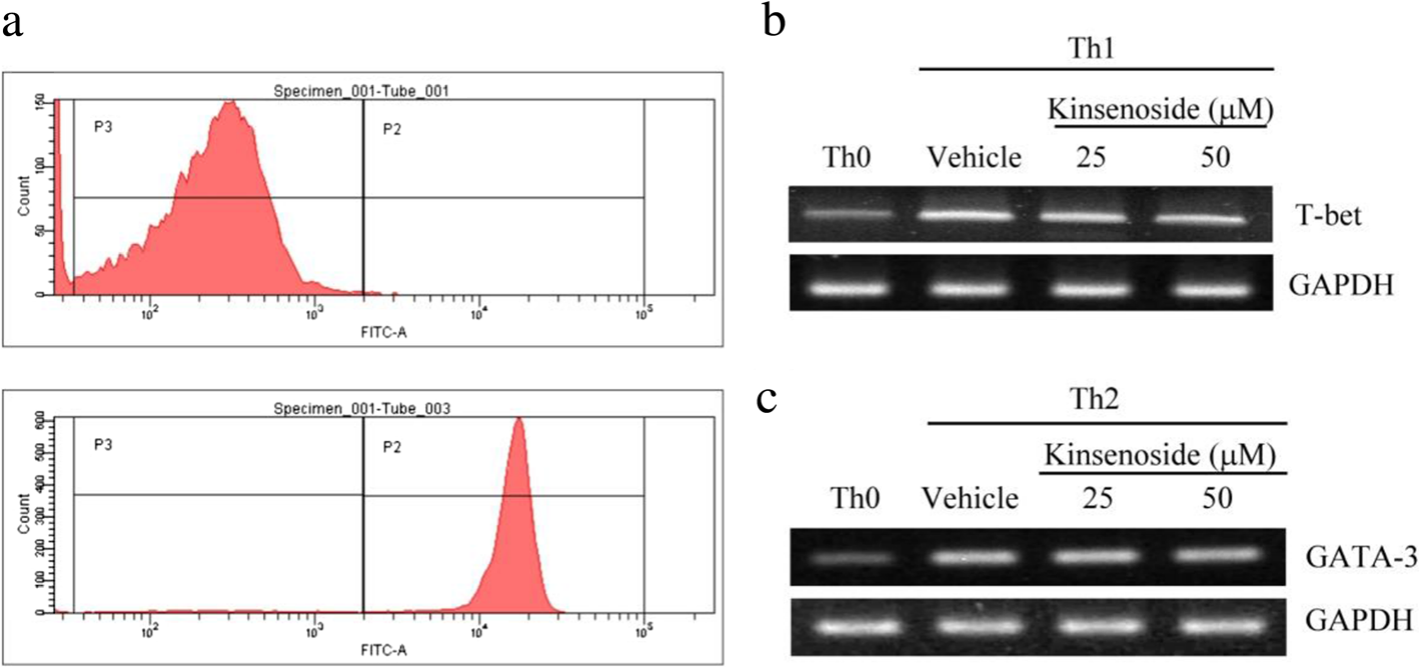
The effect of kinsenoside on helper T-cell differentiation in Th1 or Th2 polarized conditions. Th0 cells (CD4^+^) were sorted by FACSAria for RT-PCR analysis. FACSAria (BD Biosciences) was used to sort Splenic CD4+ cellswith the purity of >95% (**a**). Gene expression was measured by RT-PCR for T-bet (**b**), GATA-3 (**c**) in polarized splenocytes and compared with non-stimulated splenocytes (day 7). RT-PCR data were normalized to GAPDH expression and presented as means ± SD, *n* = 3 in each group. *##P < 0.01*as compared with the vehicle group

We investigated whether kinsenoside would produce a similar effect under Th1- and Th2-polarized conditions. CD4^+^ T cells were activated using anti-CD3/anti-CD28 and cultured in kinsenoside-containing medium under Th1-inducing or Th2-inducing conditions. To investigate the direct effect of kinsenoside on Th1- and Th2-specific cytokine gene expression, we performed real-time RT-PCR by using RNA from activated CD4^+^ T cells. Our data (Fig. 6) indicated that kinsenoside did not affect T-bet expression in the Th1-polarized culture, or GATA-3 expression in the Th2-polarized culture.

## Discussion

In this study, we demonstrated the antiinflammatory and antiarthritic effects of kinsenoside on mice with CIA, which were used as an experimental model of RA. We performed this study to elucidate the effects and modulation of kinsenoside on a CIA model. In the CIA-induced animal model, kinsenoside improved the severity of arthritis, inhibited the progression of CIA, and increased IL-10 levels. Kinsenoside markedly inhibited clinical signs of joint swelling, and significantly reduced inflammatory cell infiltration and bone erosion. These results indicated that kinsenoside exerts a distinct preventive effect on CIA.

CIA has been widely used as an animal model in RA research. It is a chronic inflammatory disease characterized by infiltration of the synovial membrane and is associated with the destruction of joints. In addition, CIA more closely resembles human RA in clinical, histological, and immunological features, as well as genetic linkage than do other experimental arthritis models [18]. Therefore, paw swelling and arthritis scores were used to measure the antiarthritic effects of various drugs on the development of the chronic inflammation of CIA. We demonstrated that the oral administration of kinsenoside produced a strong antiarthritic effect capable of reducing CIA paw edema and swelling, which was indicated by the arthritis score. RA-induced inflammation mainly occurs in the synovium and joints, which can be intuitively deduced from the results of histopathological assays. Histologically, we observed significant attenuation in the cellular infiltration of the synovium, synovial hyperplasia, and cartilage damage in mice treated with kinsenoside. In a previous study, inflammatory cytokines induced cell infiltration in joints and increased osteoclast differentiation from monocytes to attenuate disease progression and joint destruction [19]. Hsiao et al. discovered that kinsenoside suppressed osteoclastogenesis [20]. Histological analysis of the joint area of mice with CIA revealed that kinsenoside treatment markedly inhibited the infiltration of inflammatory cells, including numerous TRAP^+^ cells (osteoclasts), as well as severe osteolytic bone destruction associated with arthritic inflammation. Kinsenoside also decreased the expression of MMP-9 and CD 68^+^ cells (macrophages) in inflamed joints. Thus, our research demonstrated that kinsenoside could protect affected joints against cartilage destruction and bone erosion, and reduced cellular infiltration and synovial hyperplasia in joints.

Chronic inflammation involves the release of several mediators that are responsible for pain, as well as the destruction of bone and cartilage that can lead to severe disability [21]. However, macrophages, the major effectors of synovitis, operate through cytokine secretion, which includes TNF-α, IL-1β, and IL-6 [22]. Macrophages influence joint inflammation and the destruction of cartilage and bone in patients with RA [23], which can regulate the production of proinflammatory cytokines, including IL-1β and IL-6 in vitro [24], and stimulate inflammatory cells to secrete MCP-1 and produce MMP-9 [2]. A recent study reported that at the site of joint inflammation, the level of MCP-1 is increased in the blood, synovial fluid, and synovial tissue of patients with RA and OA, primarily by monocytes and lymphocytes [25]. The role of MCP-1 in an inflamed RA joint may be the recruitment of mononuclear phagocytes. Cytokine and chemokine are responsible for the migration and accumulation of leukocytes at inflammatory sites. MMP-9 plays a crucial role in the migration of macrophages in RA [26]. Upregulating the production of MMP-9 could degrade the cartilage matrix by stimulating proinflammatory cytokines [27]. Researchers have recognized that MMP-9 plays an essential role in the degradation of connective tissue components in the cartilage of patients with RA. The expression and production of MMP-9 is regulated at the transcriptional level by proinflammatory cytokines [28]. A reduction in disease severity and bone resorption could be caused by the blockage of these molecules [29], whereas IL-4 and IL-10 possess potent antiinflammatory characteristics and can suppress cartilage and bone pathology in patients with RA [30]. In the present study, we demonstrated that kinsenoside significantly decreased the CIA-augmented gene expression of TNF-α, IL-1β, MMP-9, and MCP-1, and increased the gene expression of IL-10 in the inflamed joints of mice with CIA. Our previous study, which demonstrated that LPS-induced gene expression of TNF-α, IL-1β and MCP-1 is inhibited and the gene expression of IL-10 is enhanced by kinsenoside in macrophages, supports these results. Therefore, we concluded that kinsenoside can suppress cartilage degradation in the inflamed joints of patients with RA.

Recent studies have demonstrated that numerous RA patients exhibit changes in morphology and inflammatory phenotypes in synovial tissue, characterized histologically by hypertrophy and the infiltration of leucocytes and monocytes or macrophages [31]. CIA has provided scientists with a translational model that defines the role of inflammatory cytokines in RA, particularly TNF-α, IL-1β, and IL-17 [32]. Therefore, RA therapy is expected to rapidly control inflammation, suppress progressive joint destruction, and be safe to use. A previous study by Hsiao et al. [11] reported that kinsenoside could effectively suppress macrophages, a critical pathway for driving proinflammatory cytokine production. Our results indicated that the administration of kinsenoside to mice with CIA inhibited IFN-γ and TNF-α production and increased IL-10 production in splenocytes by stimulating Con A production. The expression of IL-17, induced mainly by Th17 cells, in the synovium has been associated with the disease severity of RA [33]. IL-17 increases the activation of synoviocytes and expression of other cytokines, thereby contributing to cartilage and bone destruction [34]. In patients with RA and in CIA models, IL-17 was detected at increased levels in joints, and elevated levels of IL-17A were observed in the inflamed synovium [35]. In this study, the administration of kinsenoside to CIA mice inhibited the secretion of IL-17, suggesting that the antiinflammatory action of kinsenoside is associated with a significant reduction in the release of IL-17. Thus, kinsenoside downregulated the production of the proinflammatory cytokines TNF-α, IFN-γ and IL-17 in the splenocytes, and increased the levels of antiinflammatory cytokine IL-10, which ameliorated the disease.

In the past, scientists have asserted that RA is a Th1-associated disorder [36], and considered Th1 cells to be the principal T-cell players in the pathogenesis of RA. One previous study reported that Th1 cells were observed in the joints of patients with RA [37]. Tregs from healthy individuals or patients with RA receiving treatment suppressed the proliferation of responder T cells and reduced IFN-γ production [38]. Additionally, one study reported that Tregs isolated from the synovial fluid or peripheral blood of patients with RA suppressed the proliferation of autologous T cells in vitro [39]. Therefore, Tregs suppress the proliferation and cytokine production of CD4 T cells through the inhibition of IL-2 transcription. Furthermore, Tregs are crucial for the suppression of potentially harmful excessive immune responses [5]. However, kinsenoside would produce a similar effect under Th1- and Th2- polarized conditions in kinsenoside-containing medium under Th1-inducing or Th2-inducing conditions. These results indicate that kinsenoside upregulates the cell population of CD4^+^CD25^+^ Tregs, thereby attenuating the progression of CIA. Our analysis, conducted using flow cytometry, demonstrated that the administration of kinsenoside reduced the cell population of CD19^+^CD45^+^ and CD4^+^Tim-3^+^, and increased the cell population of CD4^+^CD25^+^ cells in splenocytes. These results suggested that kinsenoside downregulated Th1-cell response, thereby mediating antiinflammatory functions. Kinsenoside might also indirectly suppress Th1 response by increasing the number of Tregs. However, the increase in the production of IL-10 in splenocytes by kinsenoside is evidence against a shift toward Th2 responses. However, the naïve CD4+ cells isolated from healthy mice provided evidence that kinsenoside did not influence Th1 or Th2 differentiation ex vivo. According to our previous data, kinsenoside was responsible for the suppression of airway hyperresponsiveness in mice who inhaled OVA through the modulation of Tregs [13]. Therefore, the inhibition of Th1 cytokines by using kinsenoside is dependent of its action by modulating Tregs. Tregs can also modulate monocyte function and directly inhibit the immunoglobulin response of B cells [40]. Our results demonstrated that kinsenoside enhanced the expression of Tregs, thereby decreasing the B cell population. T-cell-dependent antigen B-cell responses exhibited similar changes and were consistent with these findings. We demonstrated that kinsenoside significantly reduced the levels of CII-specific IgG1 and IgG2a Abs in the serum of mice with RA. Kinsenoside treatment caused a deficiency in the generation of deleterious autoimmune antibody responses, suggesting that kinsenoside played an essential role in regulating B cell trafficking and antibody production.

## Conclusion

We confirmed that kinsenoside inhibits the inflammatory mediator on RA in our in vivo study. Kinsenoside effectively mitigated the destruction of cartilage and inflammatory responses in mice with CIA. These therapeutic effects partially resulted from the direct suppression of MMP-9 production in the joints by inhibiting inflammatory cytokines, including IL-1β and TNF-α. These results suggest that kinsenoside inhibits the inflammatory mediator release in a CIA induced arthritis mouse model by regulating the T cells responses.
